# Formation of porous Ga oxide with high-aspect-ratio nanoholes by anodizing single Ga crystal

**DOI:** 10.1038/s41598-023-39624-2

**Published:** 2023-07-31

**Authors:** Toshiaki Kondo

**Affiliations:** Department of Mechanical Systems Engineering, Aichi University of Technology, 50-2 Manori, Nishihasama-Cho, Gamagori, Aichi 443-0047 Japan

**Keywords:** Synthesis and processing, Corrosion

## Abstract

I developed a simple crystal growth process to obtain a single Ga crystal. The crystal orientation of a Ga plate could be controlled by a crystal growth process using a seed Ga crystal. By anodizing a [100]-direction highly oriented Ga plate, I realized the formation of a highly ordered array of high-aspect-ratio straight nanoholes. It was observed that the nanohole growth direction depends on the crystal orientation of a Ga plate. To date, this dependence has yet to be observed in materials other than porous Ga oxide obtained by an anodization process. The present fabrication process is expected to be applied to the fabrication of various functional devices requiring a porous Ga oxide with high-aspect-ratio straight nanoholes, such as hydrogen formation devices and functional filters.

## Introduction

A fabrication process for a semiconductor nanohole array with high aspect ratio has attracted attention owing to several characteristic features of such an array, namely, large surface area, anisotropic carrier transfer, and efficient material exchange in nanoholes originating from its straight shape. Owing to these characteristic features, the semiconductor nanohole array is expected to be applied to the fabrication of various functional devices, such as sensing devices, dye-sensitized solar cells, and hydrogen formation devices^1–4^. Until now, various fabrication processes for semiconductor nanohole arrays, such as reactive ion etching, metal-assisted chemical etching, and anisotropic anode etching, have been proposed^5–8^. Among them, the anodization process has attracted considerable attention owing to its simplicity, high productivity, and also the wide applicability of the obtained porous structures^9^. A metal or semiconductor is immersed in an acidic electrolyte. Basically, by applying a voltage between the metal or semiconductor and a counter electrode, an oxide nanohole array is formed on the surface of the metal or semiconductor. The formation area of a nanohole array can be easily increased by increasing the size of a material. Several materials, such as Zr, W, and Fe, are considered suitable for the fabrication of semiconductor nanohole arrays by the anodization process^10–12^. Especially, porous titania, that is obtained by anodizing Ti, is well known as a typical semiconductor nanohole array with high-aspect-ratio nanoholes^12–14^, and it has been proposed to be applied to the fabrication of energy conversion devices^14–16^.

Recently, Ga oxide has attracted attention owing to the wide applicability of its unique semiconductor properties, such as its wide band gap and appropriate redox potential for water splitting. Therefore, Ga oxide is expected to be applied to high-power electronics and hydrogen formation devices^17,18^. Ga is one of the main elements and belongs to the boron group including Al, which is a typical metal used for forming a nanohole array structure having high-aspect-ratio features by the anodization process. Analogously to Al, Ga is considered to be a candidate material suitable for the preparation of a high-aspect-ratio nanohole array by the anodization process. Until now, only a few reports on Ga anodization have been published^19–21^. In those reports, the formation of a porous Ga oxide by anodization and its application to hydrogen formation devices were discussed. However, the formation of a porous Ga oxide consisting of high-aspect-ratio nanoholes has not been reported yet. In addition, the number of reports on an anodic porous Ga oxide is still small in spite of its usefulness of the semiconductor properties. This is because of the difficulties of handling Ga originating from its low melting point of 30 °C. A single crystal is frequently prepared for anodization experiments at an early stage of understanding the anodization behavior of materials; however, the process of preparing a single Ga crystal and controlling its crystallinity have also not been established yet.

The performance of functional devices based on semiconductor nanohole arrays depends on the geometrical structures of nanoholes, which are the arrangement and aspect ratio of the nanoholes. To improve the performance of such devices, the control of the geometrical structures of the nanoholes is important. The advantageous point of applying the anodization process to the fabrication of nanoholes is the good controllability of the geometrical structures of the nanoholes. To date, the anodization process based on a pretexturing process has been proposed to control the geometrical structures of nanohole arrays^22,23^. Prior to anodization, an ideally ordered array of nanometer-scale concaves is formed on the surface of a metal or a semiconductor by a texturing process. During anodization, each concave acts as a starting point of nanohole formation, and then, an ideally ordered nanohole array is formed. Recently, the pretexturing process has been applied to the anodization of various materials, such as W, Zr, and stainless steel^24–26^. Our group has reported that a porous Ga oxide with an ideally ordered nanohole array could be obtained by the anodization process based on the pretexturing process^27^. However, a porous Ga oxide with high-aspect-ratio features could not be obtained because the arrangement of nanoholes was disordered along with the growth of nanoholes.

In the present research, a fabrication process allowing the formation of a porous Ga oxide with high-aspect-ratio straight nanoholes was investigated. In the case of anodizing Al, to understand the mechanism of anodizing Al, a highly crystalline Al was prepared and anodized. It has been reported that the formation rate of a nonporous (barrier-type) alumina layer depends on the crystal orientation of Al^28^. Analogously to this, in the case of anodizing Ga, it is expected that the formation behavior of nanoholes will depend on the crystal orientation of Ga. Therefore, I developed a simple preparation process for a single Ga crystal and a method of controlling its orientation. Then, I demonstrated the fabrication of a porous Ga oxide with high-aspect-ratio straight nanoholes by anodizing a single Ga crystal.

## Results and discussion

**Figure 1:**
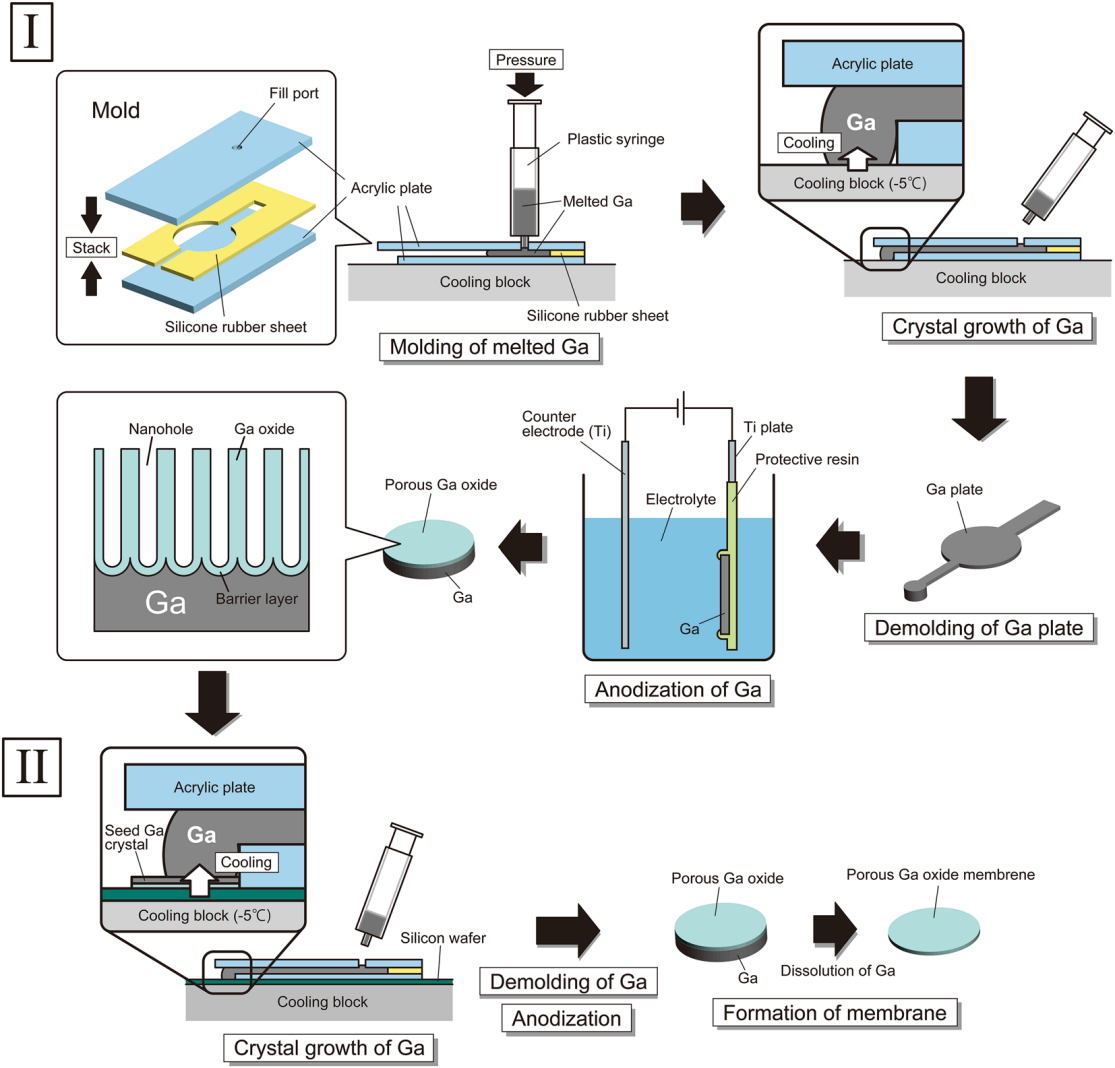
(**I**) Preparation process for a seed Ga crystal. Liquid Ga was introduced from a fill port of a mold using a syringe and filled up the mold. Liquid Ga was solidified by cooling (− 5 °C) on a Peltier device. The solidified Ga was detached from the mold. The obtained Ga plate was glued onto a Ti plate and anodized in phosphoric acid solution. (**II**) Fabrication process for a porous Ga oxide with high-aspect-ratio straight nanoholes. Liquid Ga was injected into the mold and contacted onto a seed Ga crystal. The mold on a silicon wafer was placed on the cooling block, and then, liquid Ga was solidified. By anodizing the detached Ga plate, one can form a porous Ga oxide. For the SEM observation of a barrier layer of the porous Ga oxide, part of Ga beneath a porous Ga oxide was selectively dissolved by wet etching.

A simple preparation process for an orientation-controlled single Ga crystal was investigated. The crystal orientation of Ga was controlled by a crystal growth method using a seed Ga crystal. Firstly, a seed Ga crystal was prepared. Figure 1I shows the preparation process for a seed Ga crystal. A mold was set on a cooling device. Then, liquid Ga was injected into the mold and contacted onto the cooling device. Liquid Ga was solidified from the contacted part. A solid Ga plate was detached from the mold. A porous Ga oxide was obtained by anodizing the Ga plate in an acidic electrolyte. Figure S1 shows typical cross-sectional scanning electron microscopy (SEM) images of a porous Ga oxide. Although all samples were anodized under the same conditions, the geometrical structures of the porous Ga oxide were different. Among them, a sample with straight nanoholes growing perpendicularly to the sample surface was selected. Part of the Ga of the selected sample was used as a seed crystal.

**Figure 2:**
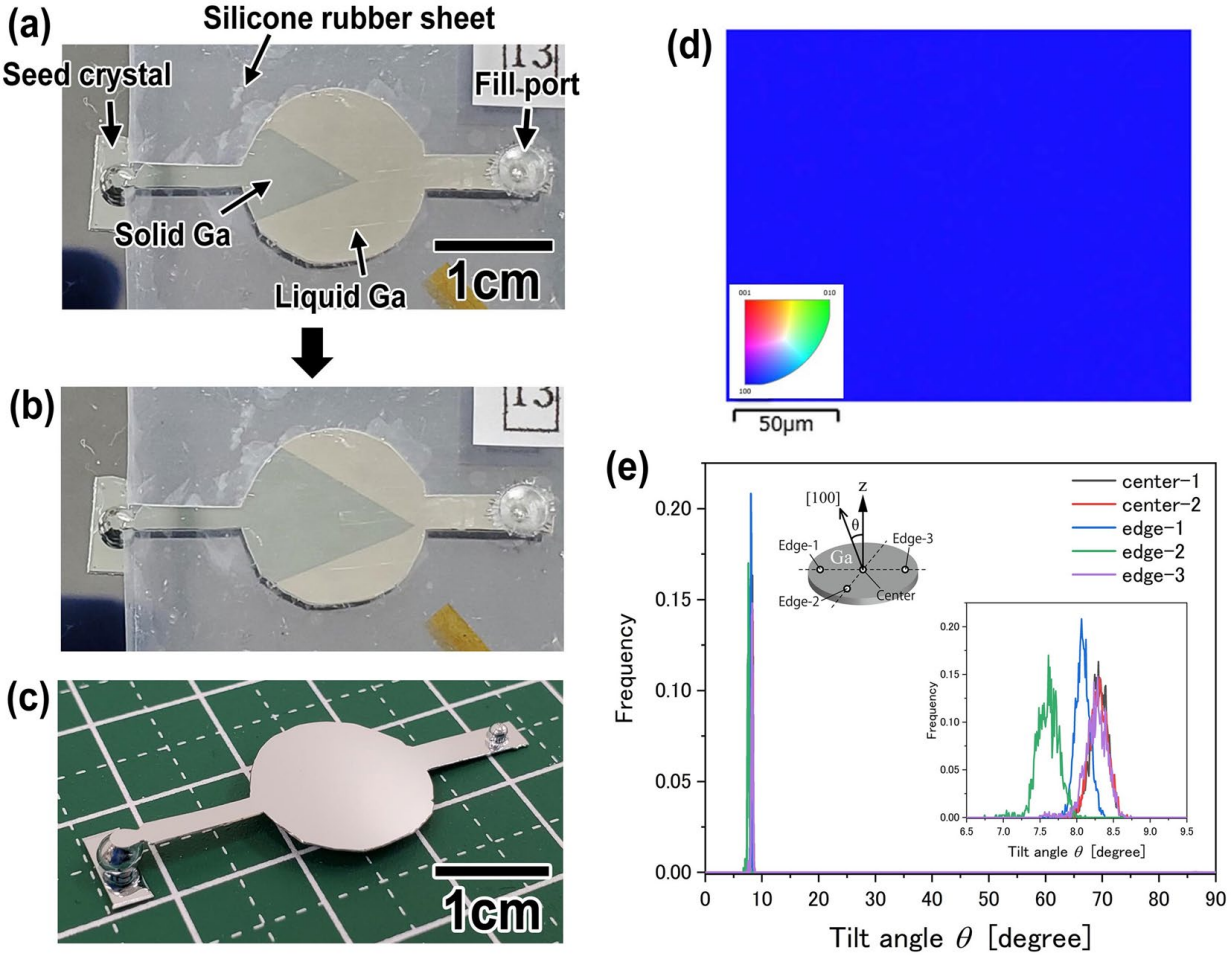
(**a**, **b**) Typical photographs of solidifying Ga. A seed Ga crystal was used for the crystal growth of Ga. The dark blue and bright silver parts correspond to solid and liquid Ga, respectively. The solidification of Ga progressed with time. (**c**) Photograph of a Ga plate obtained by the present process. (**d**) Typical EBSD image taken around the center of the Ga plate. (**e**) Tilt angle of [100] direction measured at different points as shown in inset diagram. The inset graph is a magnification of the peaks in (**e**).

A crystal-orientation-controlled Ga plate was prepared by the process shown in Fig. 1II. Liquid Ga was injected into the mold and contacted onto the seed Ga crystal, and then cooled. Liquid Ga was solidified from the part contacted onto the seed crystal. Figure 2a shows a photograph of the solidifying Ga. In Fig. 2a, the dark blue and bright silver parts correspond to solid and liquid Ga, respectively. The solid and liquid Ga parts configurated a linear boundary. The crystal growth rate usually depends on the crystal face of materials. Therefore, it is expected that the crystal face of Ga will be uniform along the boundary. As shown in Fig. 2b, the position of the linear boundary moved with time, and the solid Ga area increased. During solidification, the linear shape of the boundary was maintained. In the end, the entire sample was solidified. These results indicate that the entire Ga sample obtained has uniform crystallinity. Figure 2c shows a typical photograph of a Ga plate obtained by the present process. The diameter and thickness of the circular part were 15 mm and around 200 μm, respectively. The circular shape of the Ga plate reflected the circular patterns of the silicone sheet constituting the mold. The thickness of the Ga plate was the same as that of the silicone sheet.

The crystallinity of the Ga plate was evaluated by the electron backscatter diffraction (EBSD) method. First, in EBSD measurement, a pseudo-Kikuchi pattern was obtained. A typical pseudo-Kikuchi pattern is shown in Fig. S2. By analyzing the pattern, the crystal system and crystal orientation are derived. In the present research, a pseudo-Kikuchi pattern was automatically analyzed using a database included in the measurement software of EBSD. The analysis results showed that the crystal structure of the obtained Ga plate was of the α type, which is classified into an orthorhombic crystal system. Figure 2d shows a typical EBSD image taken around the center of the sample. The inset shows the color key. It was observed that the image appeared blue throughout the measurement area. This indicates that the [100] direction of the crystal was highly oriented in the z-axis direction, which is perpendicular to the sample surface. Figure 2e shows a graph of the tilt angle of the [100] direction from the z-axis measured at different points on a sample. Although measurements were carried out at different points, tilt angles were almost equivalent. The inset graph shows the magnification of the peaks in Fig. 2e. From this graph, the average tilt angle was determined to be 8.1°. The variation of peak position was less than 1°. The full width at half maximum (FWHM) of each graph was less than 0.5°. In addition, as shown in Fig. S3, the [010] and [001] directions were also highly oriented in the x- and y-axis (planer) directions of the Ga plate, respectively. The formation of an orientation-controlled single Ga crystal was successfully achieved by the present crystal growth process.

**Figure 3:**
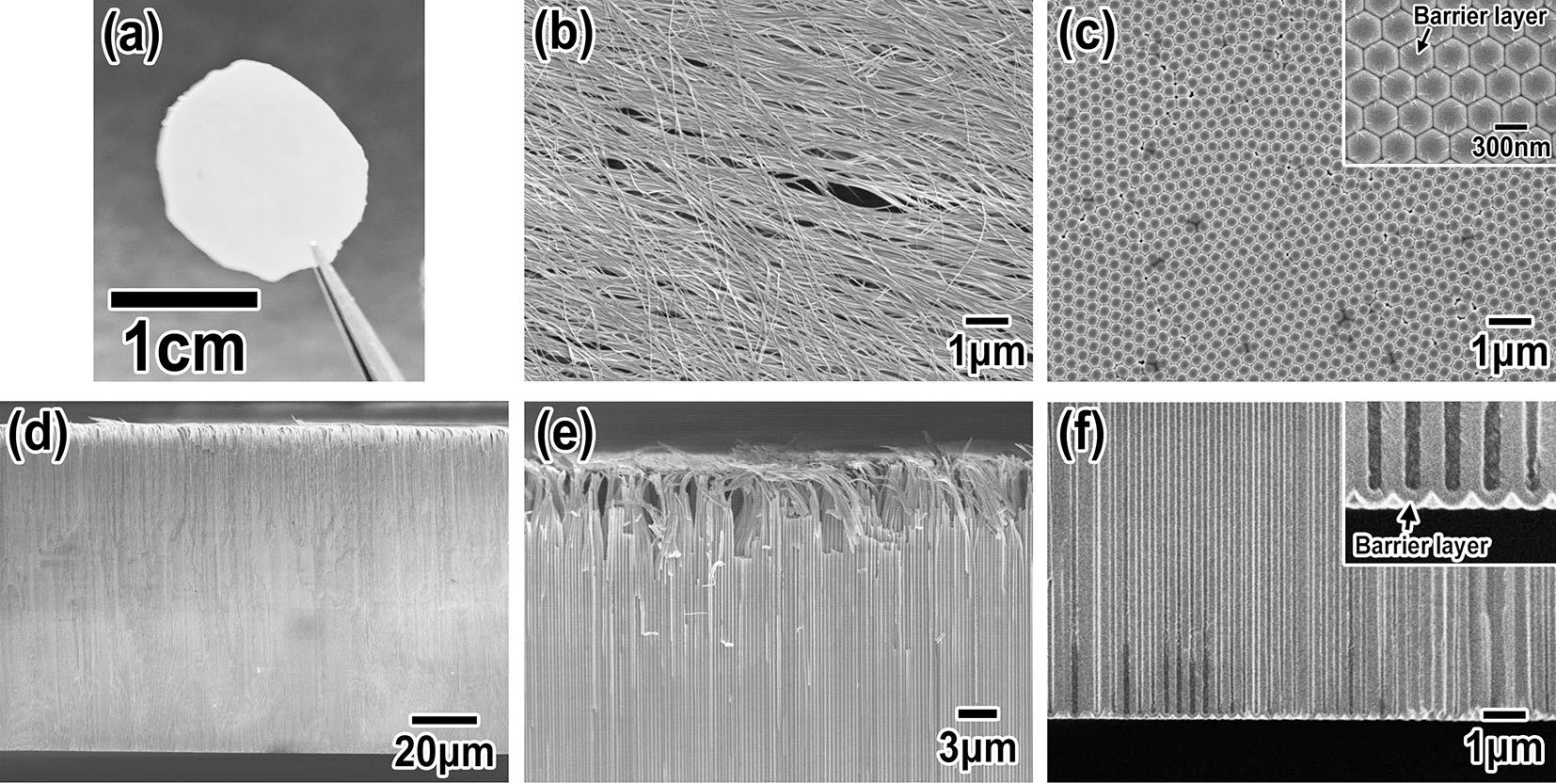
(**a**) Photograph of an obtained porous Ga oxide membrane. SEM images of (**b**) front and (**c**) back sides of the membrane. (**d**) Cross-sectional low-magnification SEM image of the membrane. The thickness of the membrane was 100 μm. High-magnification images of area around the surface of (**e**) front and (**f**) back sides of the membrane. Insets of (**c**) and (**f**) show high-magnification images of the bottom of the nanoholes.

In accordance with the procedure shown in Fig. 1II, a free-standing porous Ga oxide membrane was prepared to study its geometrical structures in detail. Figure 3a shows a typical photograph of a porous Ga oxide membrane. The porous Ga oxide layer was formed by anodizing the single Ga crystal plate for 6 h. The diameter of the membrane was 15 mm. This membrane could be easily handled by hand and tweezers. Figure 3b shows a SEM image of the front side of the membrane. The formation of Ga oxide nanowires was observed throughout the entire sample. In previous works concerning the anodization of Al, a one-step formation process for alumina nanowires has been reported^29,30^. Anodic porous alumina usually has a dual layered structure^31,32^. The outer layer, which faces the electrolyte, is usually contaminated by anions originating from the anodization electrolyte and easily dissolves into the anodization electrolyte during anodization. The inner layer, which is formed on a metal-oxide interface, consists of pure alumina. The dissolution speed of pure alumina is lower than that of contaminated alumina. In addition, the thickness of nanohole walls is relatively large at a part between three neighboring nanoholes. Even after a long time anodization, the thicker parts of the nanohole walls tend to remain, resulting in the formation of alumina nanowires. The obtained Ga oxide nanowires were considered to be formed by a similar mechanism to the formation of alumina nanowires. The diameter of Ga oxide nanowires was 70 nm. Figure 3c shows a SEM image of the another back side of the membrane. The inset in Fig. 3c shows a high-magnification image. The formation of a hemispheried structure called the barrier layer at the bottom of each nanohole was observed^26^. The observed hemispherical structures were highly ordered and constituted the domain architecture. It is considered that the highly ordered arrays of hemispherical structures were formed because the nanoholes were automatically arranged during anodization. The diameter of a hemispherical structure, which is equivalent to the interval between two neighboring nanoholes, was 320 nm. Figure 3d shows a cross-sectional SEM image of the membrane. The thickness of the membrane was 100 μm. Figure 3e shows a cross-sectional SEM image of the part around a sample surface. The formation of the nanowires was confirmed around the sample surface. Beneath the nanowires, the formation of straight nanoholes was observed. Figure 3f shows a cross-sectional SEM image of the area around the bottom of the nanoholes. Although deep nanoholes were formed after a long anodization, the straight nanohole shape was maintained without nanohole branching. It was observed that the diameter of the nanoholes around the sample surface was larger than that around the bottom of the nanoholes. A porous Ga oxide around the sample surface was formed in the early phase of anodization. The nanoholes formed early were consequently immersed in the electrolyte longer than those formed at the end of anodization. The dissolution of the walls of the nanoholes around the sample surface progressed compared with that around the bottom of the sample, resulting in the difference in nanohole diameter. The diameters of the nanoholes around the surface and bottom of the sample were 200 nm and 120 nm, respectively. The average nanohole diameter was 160 nm. The aspect ratio of the nanoholes was 625, which was derived from the average nanohole diameter of 160 nm and the hole depth of 100 μm. This is the first report on the formation of a porous Ga oxide with such high-aspect-ratio straight nanoholes. As a formation process of such high-aspect-ratio semiconductor nanoholes, an anisotropic anode etching process is widely known. The high-aspect-ratio semiconductor nanoholes could be formed owing to difference in etching speed on the crystal orientation of materials, such as GaAs^8^. However, the etched nanoholes are never automatically arranged, which is different from the case of anodization. The present anodization process is considered to have advantages for improving not only the aspect ratio of semiconductor nanoholes but also the regularity of the nanohole arrangement.

The composition of the porous Ga oxide film was evaluated by energy-dispersive X-ray spectroscopy (EDX), and quantitative analysis was carried out. In the porous Ga oxide film, the ratio of the number of Ga atoms to that of O atoms was 1:2.9. Moreover, a small number of P atoms were detected in the porous Ga oxide film. The atomic ratio of P atom was 2%. This is because the phosphorate anions originating from the anodization electrolyte were incorporated into the porous Ga oxide during anodization^20,27^.

**Figure 4:**
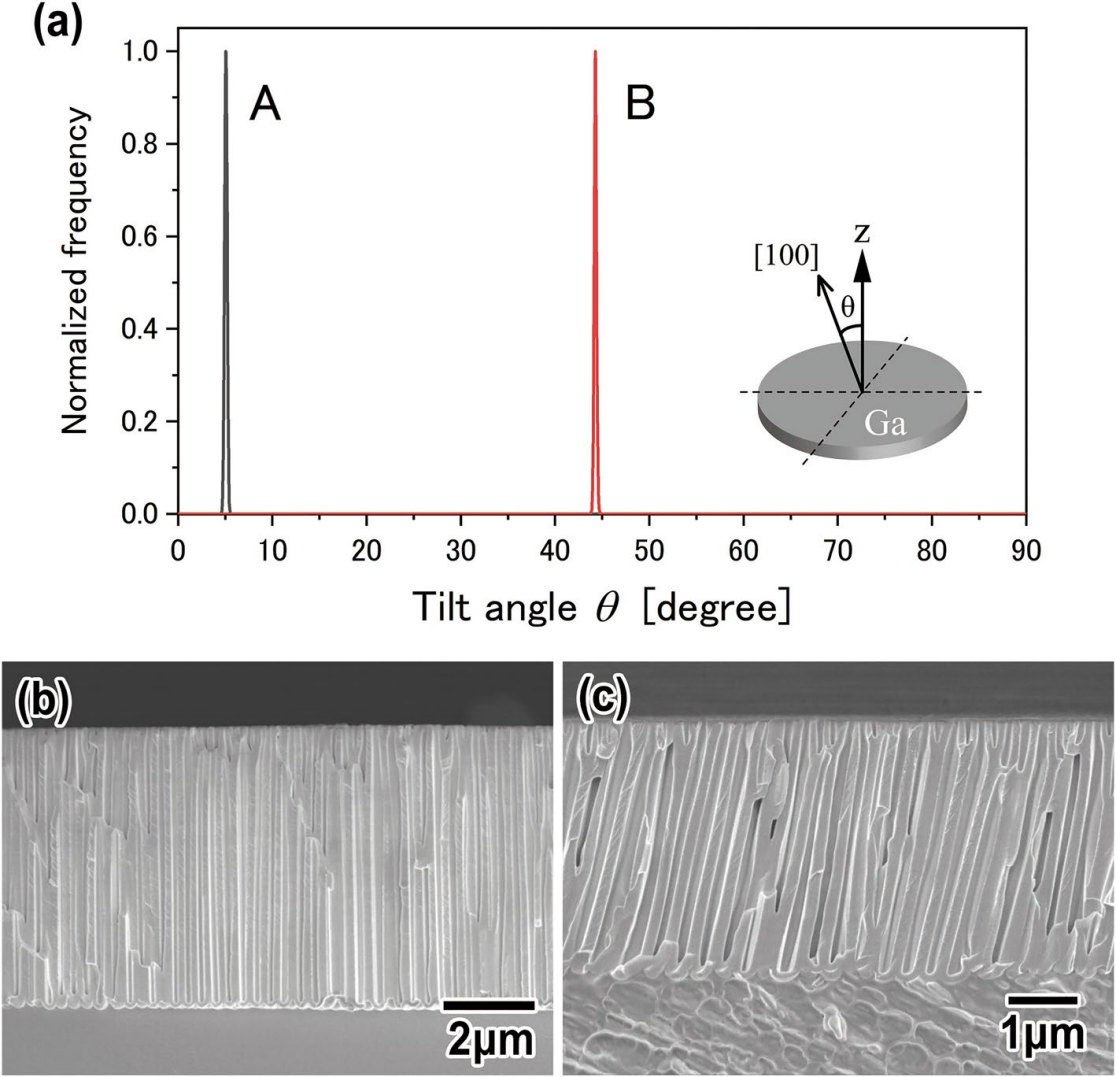
(**a**) Tilt angle of [100] direction of α-Ga from a direction perpendicular to a sample surface. The tilt angles were (A) 5° and (B) 44°. (**b**, **c**) Cross-sectional SEM images of porous Ga oxide obtained by anodizing Ga plates (A) and (B). Ga plates (A) and (B) were anodized in 1 M phosphoric acid solution at − 2 °C by applying a voltage of 80 V for 15 min. The tilt angles of the growth direction of nanoholes were (A) 1° and (B) 11°.

The dependence of the nanohole growth direction on the crystallinity of a Ga plate was investigated. First, a lot of single Ga crystal plates were prepared by the process shown in Fig. 1I. The crystal orientation of the Ga plates was evaluated by EBSD, and Ga plates (A) and (B) with different crystal orientations were selected. Figure 4a shows a graph of the tilt angles of the [100] direction of Ga plates (A) and (B). From the graph, the tilt angles of the [100] direction were 5° and 44° for Ga plates (A) and (B), respectively.

Figure 4b, c show SEM images of the porous Ga oxide obtained by anodizing Ga plates (A) and (B), respectively. From Fig. 4b, the formation of straight nanoholes that grew perpendicularly to the sample surface was observed. From Fig. 4c, the nanoholes grew obliquely. The tilt angles of the growth direction of the nanoholes were (A) 1° and (B) 11°. From the SEM images, the thickness of a porous Ga oxide layer of Ga plate (A) was larger than that of Ga plate (B). The difference in thickness can be confirmed from the difference in electrical quantity derived from the current–time curves of Fig. S4. To date, it has been reported that the formation rate of the anodic oxide layer depends on the crystallinity of materials^28^. On the other hand, the growth direction of the nanoholes was considered not related to the crystallinity of the materials for anodization. The nanoholes were usually considered to have grown perpendicularly to the sample surface. This is a first report indicating the dependence of the growth direction of straight nanoholes on the crystalline orientation of a Ga. There is a correlation between the crystal orientation of Ga and the growth rate and direction of nanoholes. Supposedly, Ga is readily anodically oxidized in the [100] direction owing to the anisotropic crystal structure of Ga, that is, an orthorhombic crystal system. The detailed mechanism of nanohole growth remains to be clarified. In future works, the dependence of the growth rate and direction of nanoholes on the crystalline orientation of Ga should be examined in detail.

## Conclusions

The present crystal growth method enabled the preparation of a single Ga crystal. The orientation of a single Ga crystal could be controlled by the present process using a seed Ga crystal. The formation of a porous Ga oxide with high-aspect-ratio straight nanoholes was achieved by anodizing a [100]-direction highly oriented Ga plate. In addition, a correlation between the growth direction of nanoholes and the crystalline orientation of Ga was observed. This correlation has yet to be observed in the anodization of metals and semiconductors other than Ga. The present fabrication process is expected to be applied to the fabrication of several functional devices requiring a porous Ga oxide with high-aspect-ratio nanoholes, such as efficient photocatalysts and functional membrane filters. In the future, if the growth direction of nanoholes is controlled by designing the Ga crystals, the present process will expand the application field of the anodization process as a novel nanofabrication process.

## Methods

### Preparation of a seed Ga crystal (Fig. 1I)

Several Ga grains (purity: 99.9999%; The Nilaco Corporation) were placed in a plastic syringe and melted by heating at 50 °C on a hotplate. A silicone sheet (thickness: 200 μm) with the desired pattern was placed between two acrylic plates (thickness: 5 mm; Sumika Acryl Sheet, Sumika Acryl Co., Ltd.). One acrylic plate has a through hole (diameter: 1.5 mm) prepared as the fill port for liquid Ga. A mold consisting of the two acrylic plates and the silicone sheet was set on a hand-made cooling device based on a Peltier element (12 V, 65 W, TEC1-12706, Stonecold). An Al plate (cooling block) on the Peltier element was cooled at − 5 °C. Then, the liquid Ga in the syringe was injected into the mold through the fill port. The liquid Ga filled up the pattern of the silicone sheet and was continuously injected into the mold. The liquid Ga overflowing from part of the mold was contacted onto the cooling block. The liquid Ga began to solidify from the contacted part. A solid Ga plate (thickness: 200 μm) was detached from the mold. A Ga plate was glued onto a Ti plate (thickness: 200 μm; The Nilaco Corporation) using a commercial conductive paste (Dotite D-500, Fujikura Kasei Co., Ltd.). Parts other than Ga were covered with protective resin (Sunecon MaskAce S, Taiyo Chemicals & Engineering Co., Ltd.). Then, a porous Ga oxide was obtained by anodizing the sample in 1 M phosphoric acid solution with stirring at − 2 °C by applying a voltage of 80 V. Anodization was carried out from 15 min to 1 h. A Ti plate was used as the counter electrode. Almost thirty samples were anodized, and geometrical structures of all samples were observed by SEM (JSM-7500F, JEOL). Among them, a sample with straight nanoholes growing perpendicularly to the sample surface was selected. Part of the Ga of the selected sample was used as a seed crystal.

### Formation process for a crystal-orientation-controlled Ga plate and a porous Ga oxide with high-aspect-ratio straight nanoholes (Fig. 1II)

At room temperature, a seed Ga crystal was set next to the acrylic mold on a silicon wafer. Liquid Ga was injected into the mold and contacted onto the seed Ga crystal. After 10 s, the mold on the silicon wafer was placed on the cooling device. Liquid Ga was solidified from the part contacted onto the seed crystal. After solidification, the Ga plate was detached from the mold. The crystal orientation of the obtained Ga plate was evaluated using an EBSD apparatus (NordlysNano; Oxford instruments) attached to a SEM system (SU-8230, Hitachi High-Tech Corporation). The obtained Ga plate was set on a Ti plate as explained above. The sample was anodized in 1 M phosphoric acid solution at − 2 °C by applying a voltage of 80 V. Anodization was carried out from 15 min to 6 h. To observe the geometrical structures of a porous Ga oxide in detail, a free-standing porous Ga oxide membrane was prepared. Part of Ga beneath the porous Ga oxide layer was selectively dissolved by immersing the sample in a saturated I_2_ methanol solution at 50 °C for 30 min. The geometrical structures of the obtained samples were observed by SEM. The composition of each sample was analyzed using EDX apparatus attached to the SEM (JSM-7500F, JEOL).

## Supplementary Information

Supplementary Information.

## Data Availability

All the data generated or analyzed during this study are included in this published article and its supplementary information files.
