# Supplementary material for: Formation of porous Ga oxide with high-aspect-ratio nanoholes by anodizing single Ga crystal
Source: Sci Rep. 2023 Jul 31;13:12408. doi: 10.1038/s41598-023-39624-2 (PMC10390530; doi:10.1038/s41598-023-39624-2)
Supplement: Supplementary file 1 — Supplementary Information. [file 41598_2023_39624_MOESM1_ESM.docx]

Supplementary


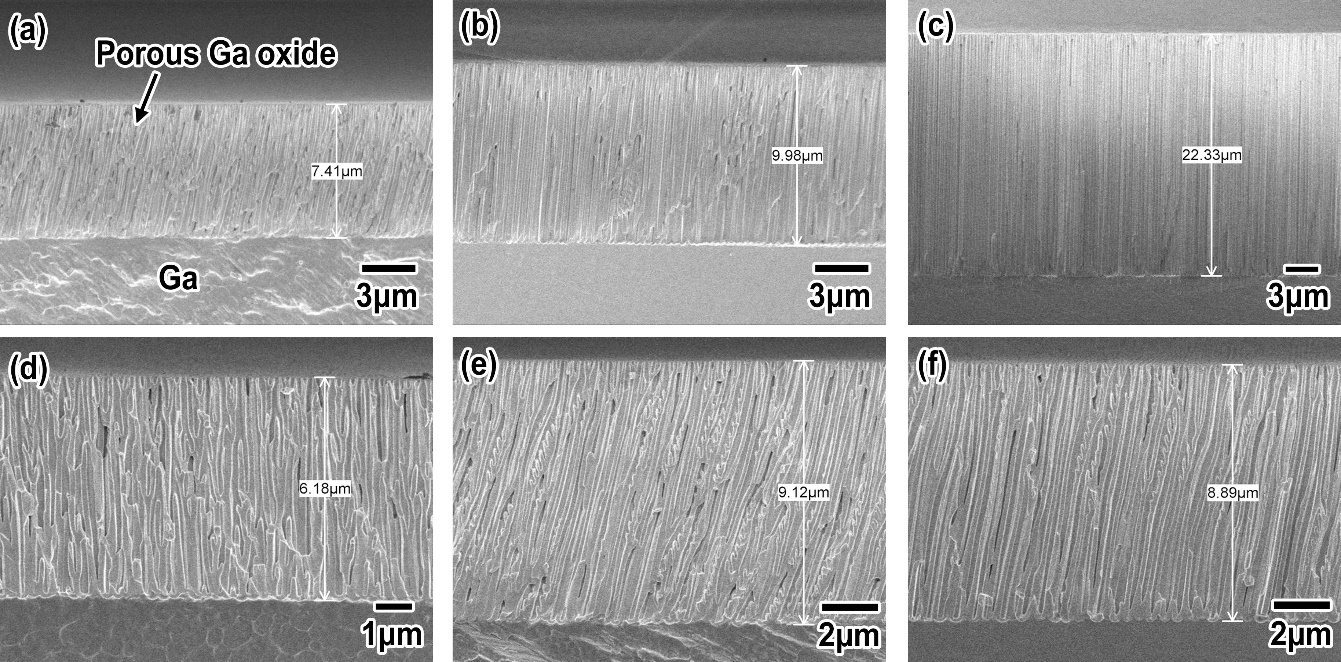


Figure S1 Cross-sectional SEM images of porous Ga oxide. All samples were obtained by anodizing Ga plates in 1 M phosphoric acid solution at -2 °C by applying 80 V for 30 min. Nanoholes grew in different directions in every sample. In the SEM image in (c), the formation of straight nanoholes growing perpendicularly to the sample surface was observed.


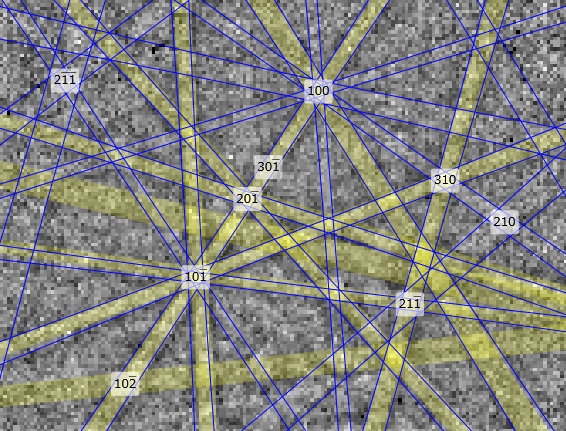


Figure S2 Typical pseudo-Kikuchi pattern of an obtained Ga plate. This pattern was captured using the EBSD software (AZtec; Oxford Instruments). Several lines consisting the pseudo-Kikuchi pattern were yellow. To determine the crystalline, more than ten yellow lines are usually required. The yellow-line pattern matched the crystalline data base in the software; then, the crystalline was automatically determined as α-Ga. The triple digit numbers on the line indicate crystal orientations.


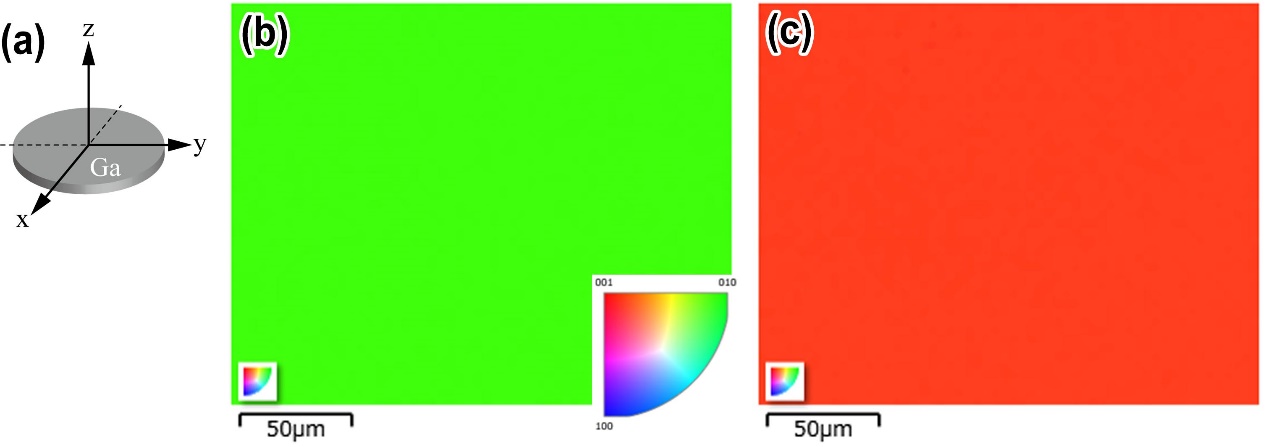


Figure S3 (a) Diagram of coordinate axes of a sample. EBSD images of a Ga plate for (b) x- and (c) y-axis directions. The inset shows the color key. [010] and [001] directions were highly oriented to the x- and y-axis directions, respectively.


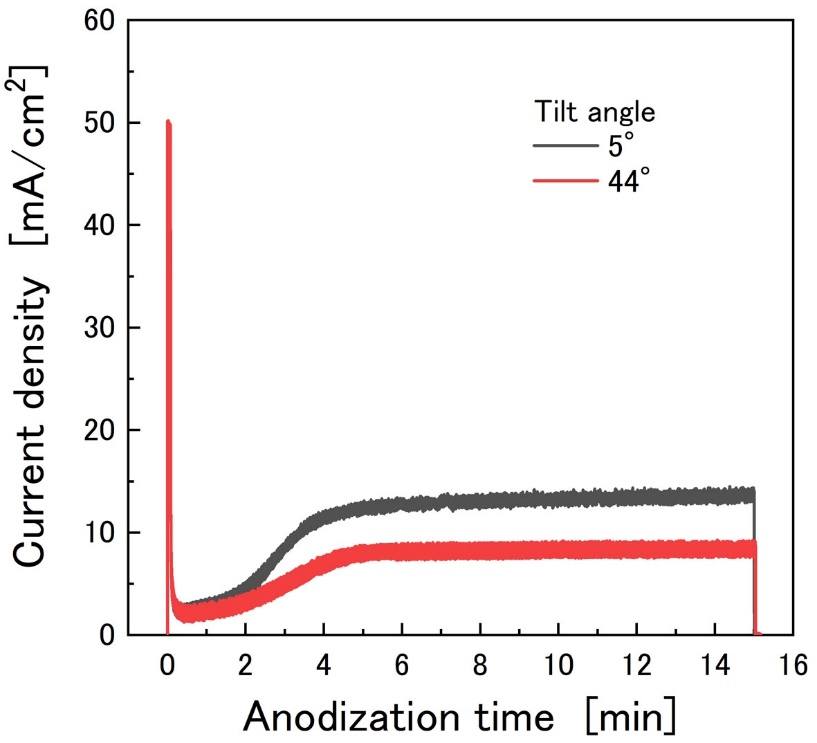


Figure S4 Current–time curves during anodization of Ga plates with different crystal orientations. The tilt angles of the [100] direction were 5° and 44°. The Ga plates were anodized in 1 M phosphoric acid solution at -2 °C by applying a voltage of 80 V for 15 min. Electric quantities were derived from the current–time curves. The electric quantities were 511 C and 325 C for the tilt angles of 5° and 44°, respectively.
